# The Two-Component System CpxRA Represses Salmonella Pathogenicity Island 2 by Directly Acting on the *ssrAB* Regulatory Operon

**DOI:** 10.1128/spectrum.02710-22

**Published:** 2022-09-08

**Authors:** Nancy León-Montes, Jessica Nava-Galeana, Diana Rodríguez-Valverde, Jorge Soria-Bustos, Roberto Rosales-Reyes, Sandra Rivera-Gutiérrez, Hidetada Hirakawa, Miguel A. Ares, Víctor H. Bustamante, Miguel A. De la Cruz

**Affiliations:** a Unidad de Investigación Médica en Enfermedades Infecciosas y Parasitarias, Hospital de Pediatría, Centro Médico Nacional Siglo XXI, Instituto Mexicano del Seguro Social, Mexico City, Mexico; b Departamento de Microbiología, Escuela Nacional de Ciencias Biológicas, Instituto Politécnico Nacional, Mexico City, Mexico; c Departamento de Microbiología Molecular, Instituto de Biotecnología, Universidad Nacional Autónoma de México, Cuernavaca, Mexico; d Unidad de Medicina Experimental, Facultad de Medicina, Universidad Nacional Autónoma de México, Mexico City, Mexico; e Department of Bacteriology, Gunma University Graduate School of Medicine, Maebashi, Japan; Centre national de la recherche scientifique, Aix-Marseille Université

**Keywords:** CpxRA, SPI-2, *ssrAB*, *Salmonella*, *cpxRA*

## Abstract

The acquisition of Salmonella pathogenicity island 2 (SPI-2) conferred on Salmonella the ability to survive and replicate within host cells. The *ssrAB* bicistronic operon, located in SPI-2, encodes the SsrAB two-component system (TCS), which is the central positive regulator that induces the expression of SPI-2 genes as well as other genes located outside this island. On the other hand, CpxRA is a two-component system that regulates expression of virulence genes in many bacteria in response to different stimuli that perturb the cell envelope. We previously reported that the CpxRA system represses the expression of SPI-1 and SPI-2 genes under SPI-1-inducing conditions by decreasing the stability of the SPI-1 regulator HilD. Here, we show that under SPI-2-inducing conditions, which mimic the intracellular environment, CpxRA represses the expression of SPI-2 genes by the direct action of phosphorylated CpxR (CpxR-P) on the *ssrAB* regulatory operon. CpxR-P recognized two sites located proximal and distal from the promoter located upstream of *ssrA*. Consistently, we found that CpxRA reduces the replication of Salmonella enterica serovar Typhimurium inside murine macrophages. Therefore, our results reveal CpxRA as an additional regulator involved in the intracellular lifestyle of Salmonella, which in turn adds a new layer to the intricate regulatory network controlling the expression of Salmonella virulence genes.

**IMPORTANCE** SPI-2 encodes a type III secretion system (T3SS) that is a hallmark for the species Salmonella enterica, which is essential for the survival and replication within macrophages. Expression of SPI-2 genes is positively controlled by the two-component system SsrAB. Here, we determined a regulatory mechanism involved in controlling the overgrowth of Salmonella inside macrophages. In this mechanism, CpxRA, a two-component system that is activated by extracytoplasmic stress, directly represses expression of the *ssrAB* regulatory operon; as a consequence, expression of SsrAB target genes is decreased. Our findings reveal a novel mechanism involved in the intracellular lifestyle of Salmonella, which is expected to sense perturbations in the bacterial envelope that Salmonella faces inside host cells, as the synthesis of the T3SS-2 itself.

## INTRODUCTION

Salmonella is a common etiological agent of gastrointestinal disease transmitted by food or water (1, 2). The genus Salmonella is composed of two species: Salmonella enterica, which comprises six subspecies, and Salmonella bongori. So far, over 2,600 different serotypes of S. enterica have been described, which can cause severe gastroenteritis and systemic infections in warm-blooded animals, including humans (3, 4). Pathogenesis of Salmonella enterica serovar Typhimurium (S. Typhimurium) is mostly due to its ability to invade and replicate within intestinal epithelial and phagocytic host cells (5). Major virulence factors of Salmonella are type III secretion systems 1 and 2 (T3SS-1 and T3SS-2) and related effector proteins, encoded in Salmonella pathogenicity islands 1 and 2 (SPI-1 and SPI-2), respectively, which were acquired by Salmonella by horizontal gene transfer events (6–8). SPI-1 and SPI-2 also encode different effector proteins, chaperones, and transcriptional regulators that are necessary for the infection by Salmonella; the effector proteins are translocated into the cytoplasm of host cells by the respective T3SS (5, 7–14).

The SPI-1 genes are expressed when Salmonella reaches the intestinal lumen, which is necessary for Salmonella invasion of epithelial cells (9–11). *In vitro*, the SPI-1 genes are expressed in the late exponential/early stationary phase of growth in nutrient-rich media such as LB, which is thought to somehow mimic the intestinal environment (SPI-1-inducing conditions) (15, 16). Once Salmonella is inside host cells, the expression of SPI-1 is repressed, whereas that of SPI-2 is activated (17–20). The SPI-2 genes are essential for the intracellular replication of Salmonella, in both phagocytic and nonphagocytic cells, in a membrane-bound niche termed the Salmonella-containing vacuole (SCV) (21). *In vitro*, the SPI-2 genes are expressed in the late stationary phase of growth in nutrient-rich media such as LB, as well as in minimal media like N-minimal medium; minimal media containing low concentrations of calcium, magnesium, and phosphate (SPI-2-inducing conditions) resemble the conditions encountered by Salmonella within host cells (15, 19, 20).

A myriad of regulators controls the expression of the SPI-1 and SPI-2 genes (1). HilD, encoded in SPI-1, is the apex of a complex regulatory cascade that activates the expression of the SPI-1 genes and other virulence genes located outside this island (22–25). On the other hand, the SsrAB two-component system (TCS) is the central positive regulator for the SPI-2 genes and other functionally related genes located outside SPI-2 (20, 26–28). The SsrAB system is encoded in the *ssrAB* operon, located in SPI-2; SsrA is the sensor kinase, and SsrB is the response regulator that directly controls the expression of target genes (1, 27). Interestingly, HilD directly induces the expression of the *ssrAB* operon, and thus the SPI-2 genes, in the late stationary phase of growth in LB (15, 29), whereas SsrB directly represses the expression of *hilD* and *hilA*, and thus the SPI-1 genes, when Salmonella is grown under SPI-2-inducing conditions or when it is inside host cells (30–32), which shows a bidirectional transcriptional communication between SPI-1 and SPI-2. In addition, the regulatory proteins SirA/BarA, HilE, and HilD form an incoherent feed-forward loop that controls the growth cost of virulence factor expression by *S.* Typhimurium (33). Additionally, the expression of *ssrAB* under SPI-2-inducing conditions is controlled by several other regulators: positively by OmpR, SlyA, and PhoP and negatively by H-NS, YgdT, and Hha (1, 28, 34–38).

CpxRA is a TCS that controls expression of virulence genes in different pathogenic bacteria (39). CpxRA is activated by signals that induce stress in the cell envelope, including protein misfolding in the periplasm, defects in peptidoglycan, elevated pH, hyperosmolarity, alterations in inner membrane lipid composition, indole, copper, ethanol, and EDTA (40, 41). CpxA is the histidine kinase that, when activated, autophosphorylates and then transfers its phosphoryl group to the aspartate residue D51 of the response regulator CpxR; in the absence of activating signals, CpxA acts as a phosphatase of phosphorylated CpxR (CpxR-P) (40–42). CpxR-P regulates the expression of target genes encoding different cellular functions or proteins with distinct activities, such as antibiotic resistance, periplasmic protein folding and degrading factors, peptidoglycan metabolic enzymes, inner membrane proteins and regulators (40, 41). CpxR can also be activated independently of CpxA, when bacteria are grown in the presence of excess carbon, such as glucose or pyruvate. This occurs through the AckA-Pta metabolic pathway, which generates acetyl phosphate from acetyl coenzyme A (acetyl-CoA) with the phosphotransacetylase (Pta) and acetate kinase (AckA) enzymes; the phosphoryl group from acetyl phosphate is transferred to CpxR (41, 42). CpxRA regulates the expression of virulence genes in enteropathogenic Escherichia coli (EPEC), uropathogenic E. coli (UPEC), enterotoxigenic E. coli (ETEC), avian-pathogenic E. coli (APEC), *Shigella* spp., Legionella pneumophila, *S.* Typhimurium, Yersinia pseudotuberculosis, and Haemophilus ducreyi (43–51). In *S.* Typhimurium, the absence of CpxA leads to the phosphorylation of CpxR through the AckA-Pta pathway; CpxR-P represses the expression of the SPI-1 and SPI-2 genes in SPI-1-inducing conditions by affecting the stability of HilD (51). A previous study reported the positive regulation of *ssrB* (the second gene of the *ssrAB* operon) by CpxR, mediated by a CpxR-binding site located between positions +19 and +51 with respect to a transcriptional start site located upstream of *ssrB* (52). However, the role of CpxRA TCS on the promoter located upstream of *ssrA* remains unknown.

In this work, we determined that the TCS CpxRA represses the expression of SPI-2 genes under SPI-2-inducing conditions by the direct action of CpxR-P on the *ssrAB* regulatory operon, specifically on the promoter located upstream *ssrA*. Two CpxR-binding sites (proximal and distal from the promoter located upstream of *ssrA*) were required for the regulation/binding of CpxRA on *ssrAB.* Consistently, our results show that CpxRA reduces *S.* Typhimurium replication within RAW264.7 macrophages. Our findings further expand the knowledge about the regulatory mechanisms controlling the intracellular lifestyle of Salmonella.

## RESULTS

### CpxRA represses *ssrAB* and SsrAB target genes.

**FIG 1:**
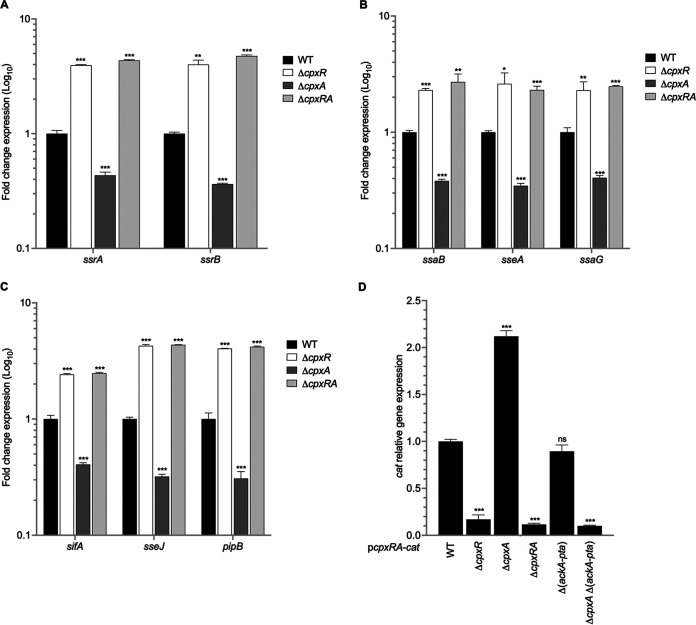
CpxRA represses expression of SPI-2 genes. Fold change in expression (RT-qPCR) of the *ssrA*, and *ssrB* genes (A), the *ssaB*, *sseA*, and *ssaG* genes (B), and the *sifA*, *sse*, and *pipB* genes (C) in the WT *S*. Typhimurium strain and its Δ*cpxR*, Δ*cpxA*, and Δ*cpxRA* mutants. (D) Fold change in expression of the *cpxRA-cat* fusion in the WT *S.* Typhimurium strain and its Δ*cpxR*, Δ*cpxA*, Δ*cpxRA*, Δ(*ackA-pta*), and Δ*cpxA* Δ(*ackA-pta*) mutants. Data are averages from at least 3 independent experiments. Error bars indicate standard deviations. ns, not significant; *, *P < *0.05; **, *P < *0.01; ***, *P < *0.001.

In order to analyze whether CpxRA controls the expression of the SPI-2 genes in SPI-2-inducing conditions, we firstly quantified by RT-qPCR the expression of the *ssrAB* regulatory operon in the wild-type (WT) *S.* Typhimurium strain and its derivative Δ*cpxR*, Δ*cpxA*, and Δ*cpxRA* mutants grown in N-minimal medium (N-MM). The expression of both *ssrA* and *ssrB* genes increased 4-fold in the Δ*cpxR* and Δ*cpxRA* mutants compared to the WT strain (Fig. 1A), suggesting that CpxR negatively regulates expression of the *ssrAB* operon. In contrast, decreases of 2- and 3-fold in the expression of *ssrA* and *ssrB*, respectively, were detected in the Δ*cpxA* mutant compared to the WT strain (Fig. 1A). Several reports have demonstrated that both null and truncated mutants in the *cpxA* gene show high levels of CpxR-P, due to the phosphorylation of CpxR by acetyl-phosphate produced by the AckA and Pta enzymes and the absence of the CpxA phosphatase activity on CpxR-P (43–48, 51). Consistently, the expression of genes positively regulated by SsrAB (*ssaB*, *sseA*, *ssaG*, *sifA*, *sseJ*, and *pipB*) was affected in the Δ*cpxA*, Δ*cpxR*, and Δ*cpxRA* mutants similarly to the expression of the *ssrA* and *ssrB* genes (Fig. 1B and C): *ssaB*, *sseA*, and *ssaG* are located in SPI-2, whereas *pipB*, *sseJ*, and *sifA* are located outside SPI-2 (14, 53). A *cpxRA-cat* transcriptional fusion was used for control of expression in N-MM. In the absence of the CpxA sensor kinase, *cpxRA* expression increased 2-fold compared to that in the WT (Fig. 1D). In contrast, *cpxRA* transcription was diminished in the Δ*cpxR* and Δ*cpxRA* mutants, corroborating the notion that *cpxRA* is positively autoregulated (54–56). While transcription of *cpxRA* was not changed in the Δ(*ackA*-*pta*) mutant, the expression of *cpxRA* was downregulated in the Δ*cpxA* Δ(*ackA*-*pta*) double mutant to levels similar to those of the Δ*cpxR* and Δ*cpxRA* mutants, showing that CpxR is constitutively phosphorylated by the AckA-Pta enzymes in a Δ*cpxA* background (Fig. 1D).

**FIG 2:**
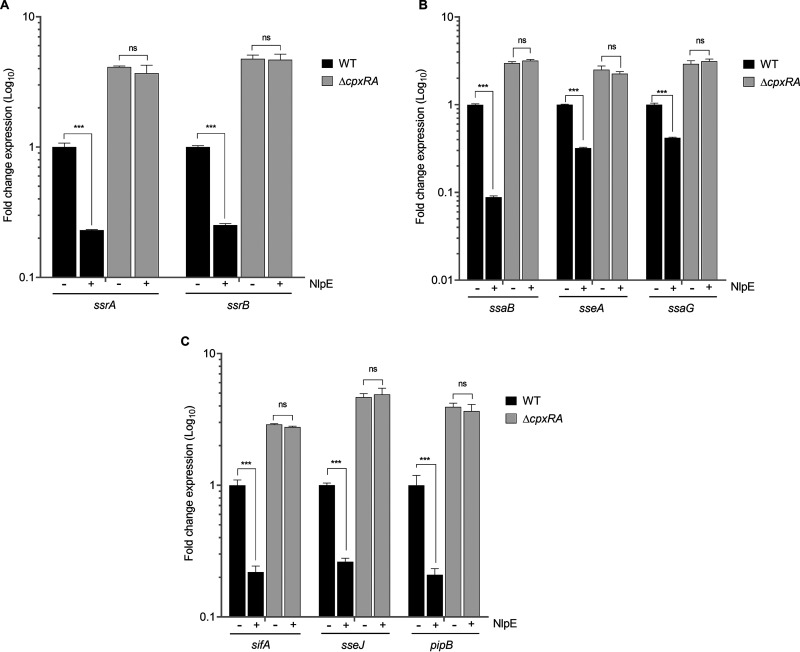
Overexpression of NlpE represses expression of SPI-2 genes through CpxRA. Fold change in expression (qRT-PCR) of the *ssrA* and *ssrB* (A), *ssaB*, *sseA*, and *ssaG* (B), and *sifA*, *sseJ*, and *pipB* (C) genes when NlpE is overexpressed in the WT and Δ*cpxRA* strains. For the induction of NlpE (+), 50 μM IPTG was added to the cultures grown in N-MM. Data are averages from at least 3 independent experiments. Error bars indicate standard deviations. ns, not significant; ***, *P < *0.001.

Overexpression of the outer membrane lipoprotein NlpE causes the activation of the TCS CpxRA (57, 58). Therefore, we evaluated by RT-qPCR the effect of NlpE-mediated activation of CpxRA on the expression of the SPI-2 genes in the WT *S.* Typhimurium strain and its Δ*cpxRA* mutant grown in N-MM. As shown in Fig. 2A, overexpression of NlpE decreased 4-fold the expression of both *ssrA* and *ssrB* in the WT strain but not in the Δ*cpxRA* mutant, corroborating that NlpE-mediated activation is CpxRA dependent (Fig. 2A). Similarly, NlpE overexpression also diminished the expression of the genes activated by SsrAB in the WT strain but not in the Δ*cpxRA* mutant (Fig. 2B and C).

Together, these results show that the CpxRA TCS represses the expression of the *ssrAB* operon and, as a consequence, the expression of SsrAB target genes under SPI-2-inducing conditions.

### *cis*-acting sequences required for the regulation of *ssrAB* by CpxRA.

**FIG 3:**
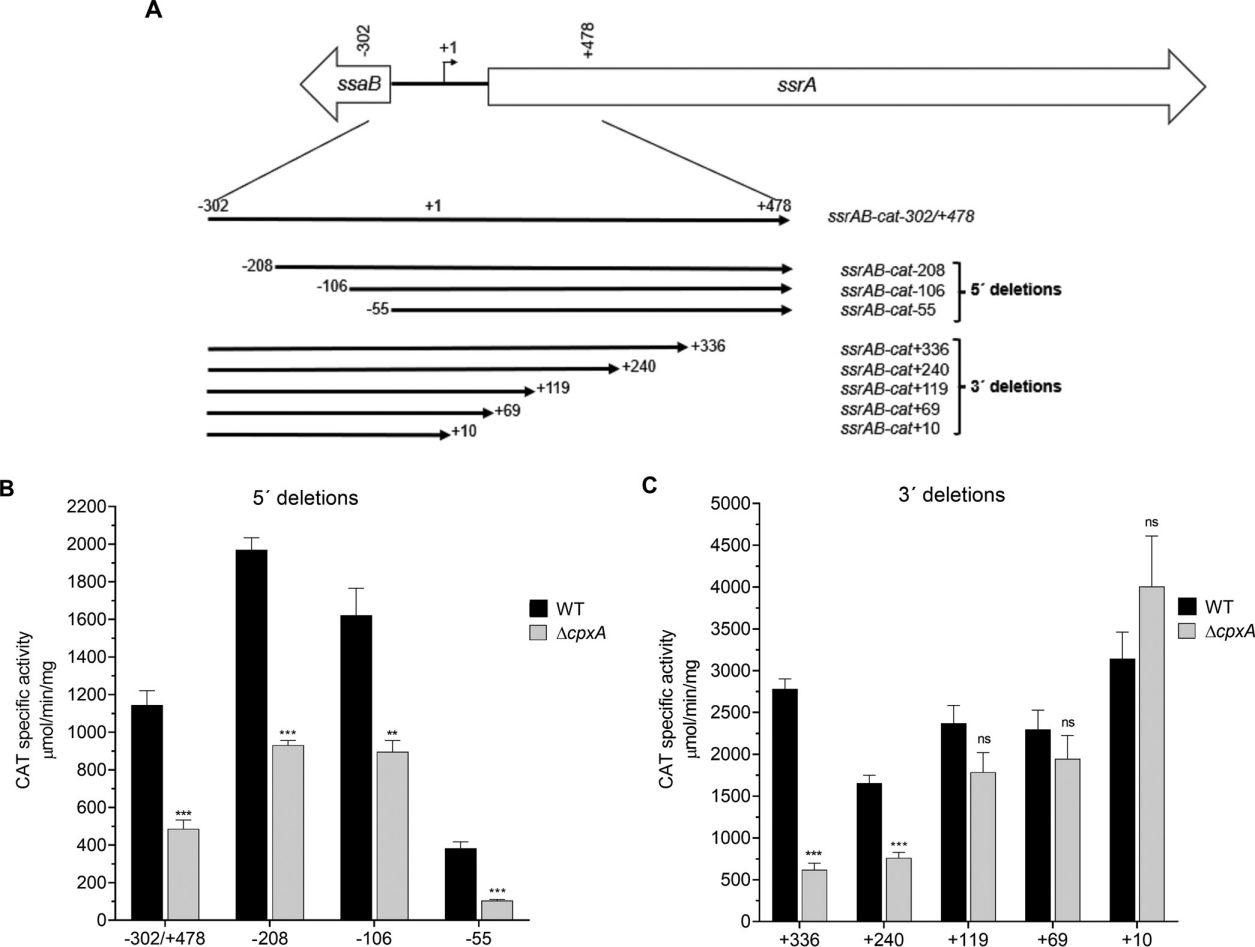
*Cis*-acting sequences required for the repression of *ssrAB* by CpxR. (A) Schematic representation of the *ssrAB* region and the *ssrAB-cat* transcriptional fusions used in this work. All positions are indicated with respect to the transcriptional start site of *ssrAB* (+1). (B and C) Expression of the *ssrAB-cat*-302/+478, *ssrAB-cat*-208, *ssrAB-cat*-106, and *ssrAB-cat*-55 (B) and *ssrAB-cat*+336, *ssrAB-cat*+240, *ssrAB-cat* +119, and *ssrAB-cat*+69 *ssrAB-cat*+10 (C) transcriptional fusions was tested in the WT *S.* Typhimurium strain and its isogenic Δ*cpxA* mutant. CAT-specific activity was determined from samples collected from bacterial cultures grown in N-MM at 37°C for 16 h. Data are averages of results from three independent experiments. Error bars indicate standard deviations. ns, not significant; **, *P < *0.01; ***, *P < *0.001.

In order to determine the *cis*-acting sequences required for the regulation of *ssrAB* by CpxRA, the expression of a series of *cat* fusions carrying different segments of the regulatory region of *ssrAB* (Fig. 3A) was quantified in the WT *S.* Typhimurium strain and its derivative Δ*cpxA* mutant grown in N-MM. The *ssrAB-cat*-302/+478 fusion, containing the most extended regulatory sequence of *ssrAB*, from position −302 to position +478 with respect to the transcriptional start site, showed decreased expression levels in the Δ*cpxA* mutant compared to the WT strain (Fig. 3B), confirming the negative regulation of CpxR on *ssrAB*. A similar expression pattern was also obtained for the *ssrAB-cat*-208, *ssrAB-cat*-106, and *ssrAB-cat*-55 fusions carrying 5′ deletions, as well as for the *ssrAB-cat*+336 and *ssrAB-cat*+240 fusions carrying 3′ deletions, their expression was decreased in the Δ*cpxA* mutant with respect to the WT strain (Fig. 3B and C). In contrast, the expression levels of the *ssrAB-cat*+119, *ssrAB-cat*+69, and *ssrAB-cat*+10 fusions carrying 3′ deletions were not significantly different between the Δ*cpxA* mutant and the WT strain (Fig. 3C). The different effects of the absence of CpxA on the *ssrAB-cat*+240 and *ssrAB-cat*+119 fusions revealed that the sequence spanning the positions +119 to +240 is required for the repression of *ssrAB* by CpxRA.

### CpxR-P binds to *ssrAB*.

**FIG 4:**
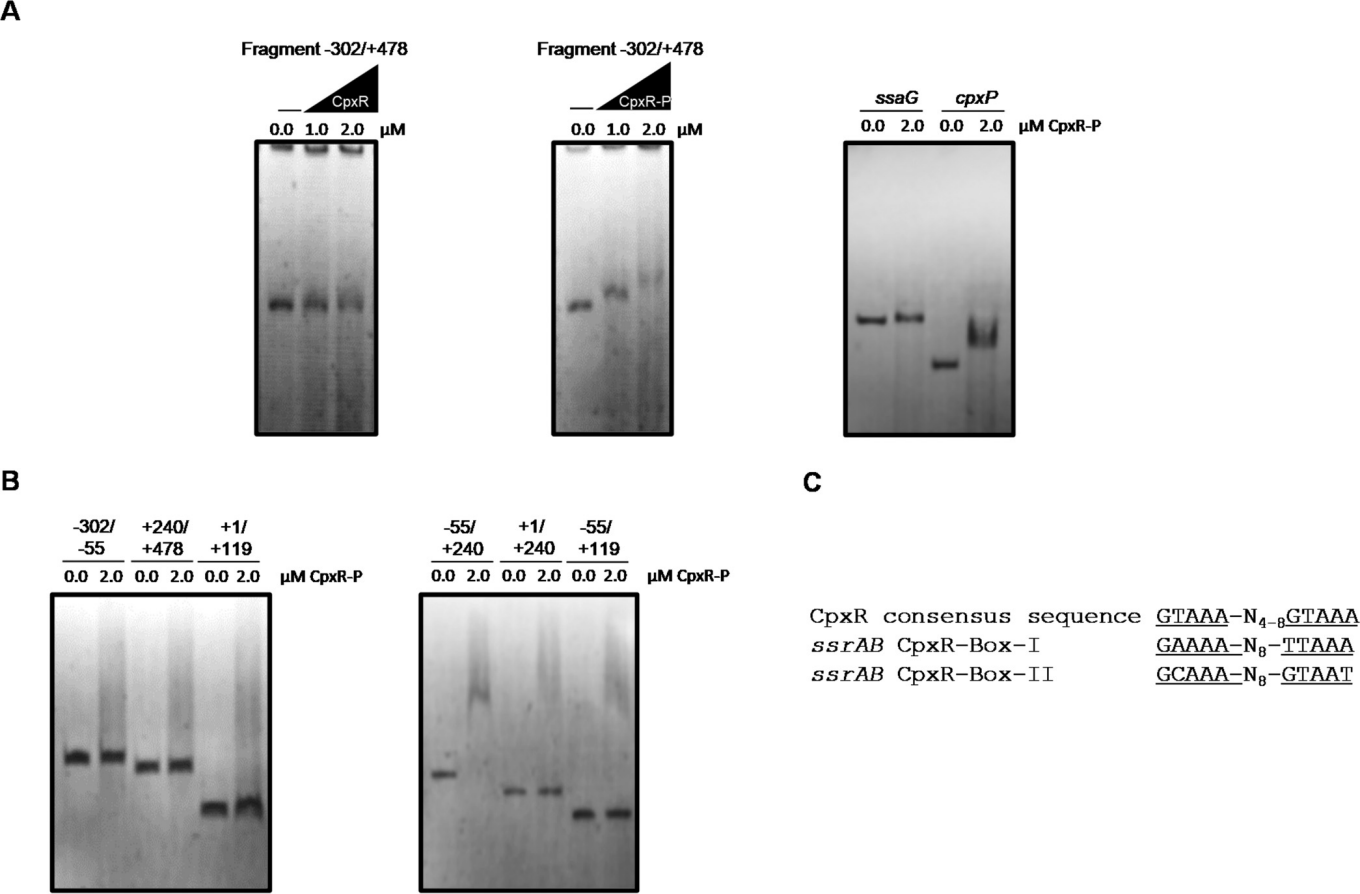
CpxR binds to the regulatory region of *ssrAB*. (A) EMSA of the DNA fragment −302/+478 with CpxR (unphosphorylated) and CpxR-P (phosphorylated) at different concentrations (0.0, 1.0, and 2.0 μM). *ssaG* and *cpxP* regulatory regions were used as negative and positive controls. (B) (Left) Binding of CpxR-P (0.0 and 2.0 μM) to sequences of the regulatory region of *ssrAB*: −302 to −55, +240 to +478, and +1 to +119. (Right) Binding of CpxR-P (0.0 and 2.0 μM) to sequences of the regulatory region of *ssrAB*: −55 to +240, +1 to +240, and −55 to +119. (C) Sequences of CpxR-binding sites on *ssrAB* regulatory region and CpxR consensus sequence for E. coli. DNA-protein complexes were resolved on a nondenaturing 6% polyacrylamide gel and stained with ethidium bromide.

To determine whether CpxRA directly regulates *ssrAB*, electrophoretic mobility shift assays (EMSAs) were performed with purified CpxR and the DNA region of *ssrAB* (fragment −302/+478) contained in the *ssrAB-cat*-302/+478 fusion, which showed regulation by CpxRA (Fig. 3B). Binding reactions were performed with phosphorylated (CpxR-P) or with nonphosphorylated CpxR. As expected, CpxR-P, but not CpxR, bound the fragment −302/+478 at concentrations of 1.0 and 2.0 μM (Fig. 4A). Additionally, CpxR-P bound the fragment carrying the upstream region of *cpxP* (positive control), a gene regulated by CpxRA (59), but it did not bind the fragment carrying the upstream region of the *ssaG* gene (negative control). Then, we analyzed binding regions of CpxR-P with different segments of the regulatory region of *ssrAB*. As shown in Fig. 4B, CpxR-P bound to a fragment spanning positions −55 to +240 of *ssrAB*. In contrast, CpxR-P did not bind fragments spanning positions −302 to −55, +1 to +119, and +240 to +478 of *ssrAB* (Fig. 4C). These results reveal that CpxR-P acts on the sequence between positions −55 and +240 of *ssrAB*. In agreement with the results from EMSAs, two putative CpxR-binding sites were manually found on *ssrAB* (boxes I and II) (Fig. 4D), according to the CpxR-binding consensus sequence [GTAAA(N)_4-8_GTAAA] reported for E. coli (60, 61). CpxR box I (GAAAAATTATTTATTAAA) and CpxR box II (GCAAACATCTTTAGTAAT) are located between positions −39 and −22 and positions +198 and +215, respectively, with respect to the transcriptional start site of *ssrA*. Interestingly, fragments containing only one of the putative CpxR-binding sites, fragment −55/+119 (carrying CpxR box I) and fragment +1/+240 (carrying CpxR box II), were not bound by CpxR-P (Fig. 4B), suggesting that both sites are necessary for binding of CpxR-P on *ssrAB*. In agreement with this conclusion, the expression of *ssrAB-cat* fusions carrying only the CpxR-I box (*ssrAB-cat*+119, *ssrAB-cat*+69, and *ssrAB-cat*+10) was not repressed by the absence of CpxA (Fig. 3C).

Thus, these results support that CpxR-P binds two sites located on the sequence spanning the positions −55 and +240 of *ssrAB*.

### CpxRA negatively affects the intracellular replication of Salmonella.

**FIG 5:**
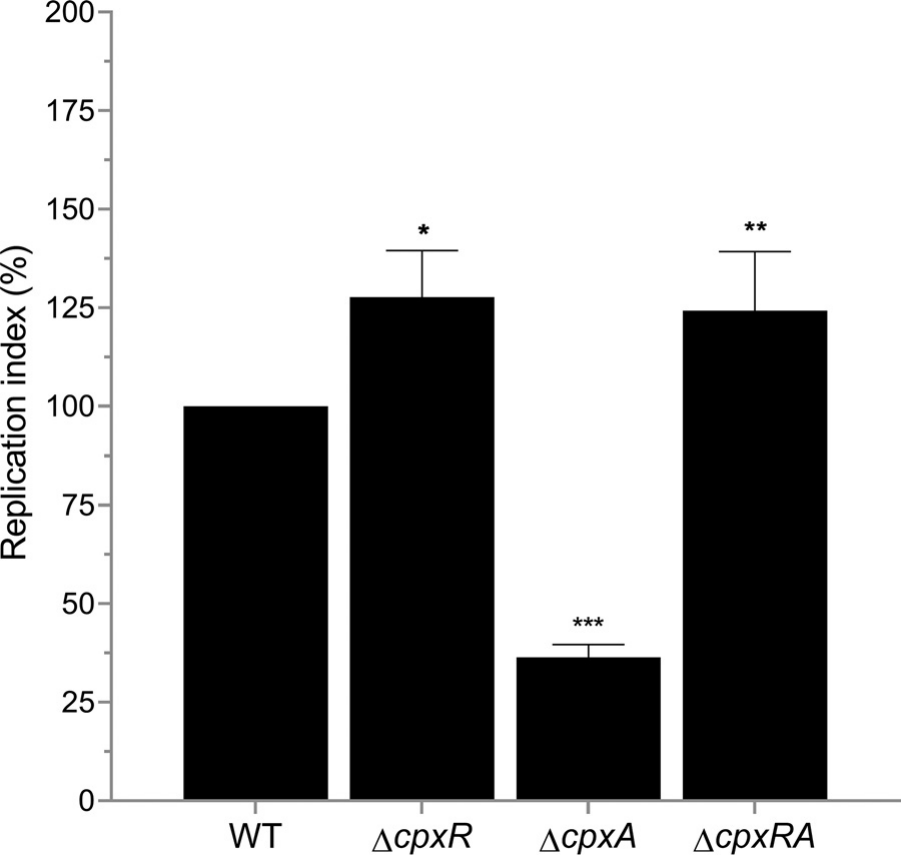
CpxRA decreases replication of *S.* Typhimurium within murine macrophages. RAW264.7 macrophages were infected with the WT *S.* Typhimurium strain and its Δ*cpxR*, Δ*cpxA*, and Δ*cpxRA* mutants. Macrophages were lysed at 2 h and 18 h to quantify intracellular bacteria. The values represent the fold increase (expressed as percentages) calculated with the ratio of intracellular bacteria between 2 h and 18 h and normalized to the value for the WT strain. Data are averages of results from three independent experiments. Bars represent standard deviations. *, *P < *0.05; **, *P < *0.01; ***, *P < *0.001.

Since our results revealed that CpxRA represses the expression of SPI-2 genes under conditions resembling the intracellular environment, we hypothesize that CpxRA negatively controls the intracellular replication of Salmonella. To investigate this, we analyzed the replication within RAW264.7 murine macrophages of the WT *S.* Typhimurium strain and its Δ*cpxR*, Δ*cpxA*, and Δ*cpxRA* mutants. As shown in Fig. 5, the intracellular replication of the Δ*cpxR* and Δ*cpxRA* mutants was ~25% higher, whereas the intracellular replication of the Δ*cpxA* mutant was ~63% lower, than that of the WT strain. Therefore, our results show that CpxR decreases the replication of *S.* Typhimurium inside macrophages, which can be explained, at least in part, by its negative control of the expression of the *ssrAB* regulatory operon.

## DISCUSSION

In this study, we found that in *S.* Typhimurium, the TCS CpxRA represses the expression of SPI-2 and functionally related genes in conditions resembling the intracellular environment (SPI-2-inducing conditions). This regulation is mediated directly on the *ssrAB* operon, encoding the TCS SsrAB, the central positive regulator for the SPI-2 and functionally related genes. Our results support that CpxR-P binds two sites located on the sequence spanning positions −55 to +240 of *ssrAB*, where two putative CpxR-binding sites were identified, at positions −39 to −22 (GAAAA-N_8_-TTAAA) and +198 to +215 (GCAAA-N_8_-GTAAT). These sequences did not show a high identity with the GTAAA(N)_4-8_GTAAA tandem repeat described for E. coli (60, 61). However, a perfect match of the consensus CpxR-binding-site on the DNA is not required, because it does not correlate with the strength of the transcription regulation (56). We determined that in the absence of the sequence between positions +119 and +240, carrying one of the identified CpxR-binding sites, the negative regulation of the *ssrAB* operon by CpxR is lost. Consistently, our results support that CpxR-P requires the two identified sites for binding on *ssrAB*. Interestingly, repression of *ssrAB* by the histone-like H-NS protein also requires the two sequences that mediates the repression of this operon by CpxR-P (29).

Subramaniam et al. reported that CpxR positively regulated *ssrB* (the second gene of the *ssrAB* operon) in the no-carbon-E (NCE) medium, finding a CpxR-binding site (GTAAA-N_5_-GGAAA) located in the sequence between positions +19 and +51 with respect to a transcriptional start site located upstream of *ssrB* (52). It is important to note that this CpxR-binding site on *ssrB* was not tested in our study. Therefore, CpxR seems to regulate the expression of *ssrAB* by acting on multiple binding sites.

The TCS CpxRA has been shown to repress virulence genes in other bacteria, such as Y. pseudotuberculosis, Shigella sonnei, *S.* Typhimurium, and different pathotypes of E. coli: enteropathogenic, enterohemorrhagic, and avian pathogenic (43–51, 62). CpxR seems to follow two different mechanisms to repress gene expression. (i) CpxR-P recognizes one site on the regulatory sequence overlapping the −35 and −10 boxes and subsequently blocks binding of RNA polymerase to the promoter. This regulation mode has been reported for the *csgBAC*, *csgDEFG*, *motAB*, *cheAW*, *rpoE*, *tsr*, *ung*, *aer*, and *rovA* genes (60, 63–65). (ii) CpxR-P binds to two or more sites on the regulatory region, competing with activators by binding to sites on the promoter and also blocking binding of RNA polymerase. This mechanism has been described for *ompF* and *papBA* genes (66, 67). The repression model of CpxR-P on *ssrAB* would imply competition between CpxR and OmpR, similar to the observed for the porin gene *ompF*, which is supported by the overlapping of two DNA-binding sites of both regulators on the *ssrAB* promoter: OmpR, −83/+6 and +130/+146 (35), and CpxR, −55/+1 and +119/+240.

To control gene expression, CpxRA respond to signals that induce extracytoplasmic stress, such as protein misfolding in the periplasm, defects in peptidoglycan, elevated pH, hyperosmolarity, alterations in inner membrane lipid composition, indole, copper, ethanol, and EDTA (40, 41). Furthermore, CpxRA seems to act as a switch-off system for the biogenesis of diverse structures localized between the inner and outer membranes, like the type III secretion systems present in many bacteria, such as S. sonnei, Y. pseudotuberculosis, enteropathogenic E. coli, enterohemorrhagic E. coli, and *S.* Typhimurium (T3SS-1 and T3SS-2) (44–47, 50, 51). Thus, the CpxRA system could be activated by misfolded structural proteins of T3SS-2 when *S.* Typhimurium resides within a late SCV.

Hha and YdgT have been described as negative regulators of SPI-2. These proteins do not bind to DNA; instead, they act as modulators of the transcription by direct interaction with H-NS (68, 69), which is the main repressor of SPI-2 under SPI-2-inducing conditions (27, 70, 71). EIIA^Ntr^, a component of the nitrogen-metabolic phosphotransferase system, also acts as a negative regulator of SPI-2; it interacts with SsrB and thus blocks DNA binding of SsrB on target genes (72). Therefore, CpxR is a repressor that directly interacts with the *ssrAB* regulatory region downregulating the promoter located upstream *ssrA*.

We found that CpxRA inhibits the replication of *S.* Typhimurium inside mouse macrophages. Interestingly, a previous study showed that the TCS PhoP/PhoQ prevents the overgrowth of *S.* Typhimurium inside nonphagocytic cells (73). Therefore, it is reasonable to think that CpxRA and PhoP/PhoQ, and possibly other regulators, such as H-NS (70, 71), EIIA^Ntr^ (72), Hha (68), and YdgT (74), coordinate to restrict intracellular overgrowth of Salmonella within host cells.

Our findings further expand the knowledge about the mechanisms mediating the intracellular lifestyle of Salmonella.

## MATERIALS AND METHODS

### Bacterial strains, media, and culture conditions.

Bacterial strains used in this study are listed in Table 1. Bacterial cultures were grown at 37°C in lysogeny broth (LB) at pH 7.5 or in N-minimal medium (N-MM) [5 mM KCl, 7.5 mM (NH_4_)_2_SO_4_, 0.5 mM K_2_SO_4_, 1 mM KH_2_PO_4_, 100 mM Tris-HCl, 10 μM MgCl_2_, 0.5% glycerol, and 0.1% Casamino Acids] at pH 7.5. Flasks of 250 mL containing 30 mL of N-MM were inoculated with bacterial suspensions prepared from overnight cultures in LB, adjusted to an optical density at 600 nm (OD_600_) of 0.05, and then incubated at 37°C in a shaking incubator at 200 rpm to an OD_600_ of 0.8 to 1.0. When necessary, media were supplemented with ampicillin (200 μg/mL), chloramphenicol (30 μg/mL), or kanamycin (50 μg/mL).

**TABLE 1:** Bacterial strains and plasmids used in this study

Strain or plasmid	Genotype or description^*a*^	Source or reference
*S.* Typhimurium		
14028	Wild type	ATCC
Δ*cpxR* mutant	14028 Δ*cpxR*::Km^r^	51
Δ*cpxA* mutant	14028 Δ*cpxA*::Km^r^	51
Δ*cpxRA* mutant	14028 Δ*cpxRA*::Km^r^	51
Δ(*ackA-pta*) mutant	14028 Δ(*ackA-pta*)::Km^r^	51
Δ*cpxA* Δ(*ackA-pta*) mutant	14028 Δ*cpxA*::FRT Δ(*ackA-pta*)::Km^r^	51
E. coli		
DH5α	E. coli K-12 laboratory strain	Invitrogen
BL21(DE3)	Strain for expression of recombinant proteins	Invitrogen
Plasmids		
pCA24N	High-copy-number cloning vector, *lac* promoter; *lacI*^q^ Cm^r^	80
pCA-NlpE	pCA24N derivative expressing E. coli K-12 NlpE from the *lac* promoter	80
pKK232-8	pBR322 derivative containing a promoterless chloramphenicol acetyltransferase (*cat*) gene; Ap^r^	81
pcpxRA-cat	pKK232-8 derivative containing a *cpxRA-cat* transcriptional fusion from nucleotides −544 to +57	51
pssrAB-cat-302/+478	pKK232-8 derivative containing a *ssrAB-cat* transcriptional fusion from nucleotides −302 to +478	29
pssrAB-cat-208	*ssrAB-cat* transcriptional fusion from nucleotides −208 to +478	29
pssrAB-cat-106	*ssrAB-cat* transcriptional fusion from nucleotides −106 to +478	29
pssrAB-cat-55	*ssrAB-cat* transcriptional fusion from nucleotides −55 to +478	29
pssrAB-cat+336	*ssrAB-cat* transcriptional fusion from nucleotides −302 to +336	29
pssrAB-cat+240	*ssrAB-cat* transcriptional fusion from nucleotides −302 to +240	29
pssrAB-cat+119	*ssrAB-cat* transcriptional fusion from nucleotides −302 to +119	29
pssrAB-cat+69	*ssrAB-cat* transcriptional fusion from nucleotides −302 to +69	29
pssrAB-cat+10	*ssrAB-cat* transcriptional fusion from nucleotides −302 to +10	29
pssaG1-cat	pKK232-8 derivative containing an *ssaG*-*cat* transcriptional fusion from nucleotides −232 to +361	15
pQE80	Vector for expression of His-tagged protein; Ap^r^	Qiagen
pQE80cpxR	N-terminal His_6_-CpxR overexpression plasmid; Ap^r^	79

a The coordinates for the *cat* fusions are indicated with respect to the transcriptional start site for each gene. Ap^r^, ampicillin resistance; Cm^r^, chloramphenicol resistance; Km^r^, kanamycin resistance.

### Quantitative RT-PCR.

Total RNA was extracted from bacteria grown in N-MM using the hot-phenol method (75). The RNA was pelleted by centrifugation at 19,000 × *g* for 10 min at 4°C. Pellets were washed three times with cold 70% ethanol and centrifuged at 16,000 × *g* for 10 min at 4°C. The ethanol was removed, the pellets were air dried for 15 to 20 min in a centrifugal vacuum concentrator (5301; Eppendorf). The RNA was resuspended in diethyl pyrocarbonate (DEPC)-treated water, DNA was removed with Turbo DNA-free (Ambion), and the quality of RNA was assessed using a NanoDrop One instrument (Thermo Scientific) and with a bleach denaturing 2% agarose gel, as previously described (76). cDNA was synthesized using 1 μg of RNA, 5 pmol/μL of random hexamer primers, and 200 U/μL of RevertAid Moloney murine leukemia virus (M-MuLV) reverse transcriptase (RT) (Thermo Scientific). Specific primers were designed with Primer3Plus software and are listed in Table 2. Quantitative RT-PCR was performed in a LightCycler 480 instrument (Roche). The absence of contaminating DNA was tested by the lack of amplification products after 45 qPCR cycles using RNA as the template. Control reactions with no RNA template and with no reverse transcriptase enzyme were run in all experiments. The relative gene expression was calculated using the 2^−ΔΔ^*^CT^* method (77). These experiments were performed in triplicate three independent times.

**TABLE 2:** Primers used in this study

Use and primer	Sequence (5′–3′)
qPCR	
*rrsH*-5′	AGGCCTTCGGGTTGTAAAGT
*rrsH*-3′	ATTCCGATTAACGCTTGCAC
*ssrA*-5′	TGATGACGTCGGCTTTTTGC
*ssrA*-3′	TTGTGCTGGTAAACGTGTGC
*ssrB*-5′	AGCGGCATTGCAAACAGTAG
*ssrB*-3′	TCGCGCAAAGTAAGCAGTTG
*ssaB*-5′	TGTGACACTACTGCTTGCAG
*ssaB*-3′	ACCGTTTAACCATCCCCCATC
*sseA*-5′	TCAACAGCTTGCTGAAAGGG
*sseA*-3′	TTAAATCCTTCTCGGCCTCCTG
*ssaG*-5′	TGGATATGCTCTCCCACATGG
*ssaG*-3′	CTGCTGTAAGGCAAATTGCG
*sifA*-5′	TGGAAAGCGCAAGAAAAGGC
*sifA*-3′	GGTGTAAAATGCGCGTTGTC
*sseJ*-5′	AGGAACACGCCGATAAGTTG
*sseJ*-3′	TGCAAGACCAAAAGCTTCCG
*pipB*-5′	ACCGCTGCAATTCTATTCGG
*pipB*-3′	CGGCTCCTGTTAATGCTTTCG
EMSA	
cpxP-5′	CATGACGGCAGCGGTAACTA
cpxP-3′	GTTTTGCTGTTGCTCGCTCC
ssaG-5′	GTAGTTTGGGACTACAGCCTC
ssaG-3′	CAATAACCGTTAGCGCTGG
−302/−55-5′	TTCGGCCCTGATATCCTGAA
−302/−55-3′	ATGTCAATGCTGAAAATGTAATTGTGA
+240/+478-5′	ACAACAATAATTATTTGGCTGCTATCT
+240/+478-3′	CCGACAGATAGATGCCGGC
+1/+119-5′	CATCGCCATCTTATTAAAAAGTAAT
+1/+119-3′	CTATCGCAGTACATATAGTTTCA
−55/+1-5′	TAAAAACTTACAATTTGAAAAATTAT
−55/+1-3′	GTAAAAACATCGTAACAGTTTATT
+119/+240-5′	GTGATCAAGTGCCAAAGATT
+119/+240-3′	TTAATAAAAATAAAAAAGTTAGCC

### CAT assays.

Chloramphenicol acetyltransferase (CAT) assays were performed as described previously (78).

### Overexpression and purification of His_6_-CpxR.

The E. coli strain BL21 transformed with the pQE80cpxR plasmid (79) was used to express and purify His_6_-CpxR. A flask containing 250 mL of LB with ampicillin (100 μg/mL) was inoculated 1:100 with an overnight culture of E. coli BL21+pQE80cpxR and then incubated at 37°C with shaking to an OD_600_ of 0.6 to 0.8 (~4 h). Subsequently, isopropyl-β-d-thiogalactopyranoside (IPTG) was added to a final concentration of 1 mM, and the bacterial culture was grown for an additional 3 h at 37°C under the same conditions. Cells were then pelleted by centrifugation and resuspended in 1× phosphate-buffered saline (PBS)–8 M urea, pH 8.0, and lysed by sonication. The lysate was centrifuged, and the soluble fraction was loaded in a nickel-nitrilotriacetic acid (Ni-NTA) agarose column (Qiagen) pre-equilibrated with lysis buffer. After 10 washes with buffer containing 50 mM imidazole (200 mL), His_6_-CpxR was eluted with 500 mM imidazole (10 mL). Purified protein was dialyzed over 2 h using a cellulose membrane (dialysis tubing, cellulose membrane; Sigma) and a buffer composed of Tris-HCl (20 mM) (pH 7.5), KCl (50 mM), dithiothreitol (1 mM), and 10% glycerol. Then, the His_6_-CpxR protein was analyzed by SDS-PAGE and Coomassie blue staining, and its concentration was determined by the Bradford procedure; it was stored at −70°C.

### EMSAs.

EMSAs were performed with purified His_6_-CpxR and the *ssrAB* fragments, which were amplified by PCR with primer pairs showed in Table 2. A DNA fragment containing the regulatory region of *ssaG*, used as negative control, was amplified by PCR using the primer pair ssaG-5′/ssaG-3′ and the pssaG1-cat plasmid as the template. A DNA fragment carrying the *cpxP* regulatory region, used as a positive control (59), was amplified by PCR using the primer pair cpxP-5′/cpxP-3′ and chromosomal DNA of E. coli MC4100 as the template. PCR products were purified using the QIAquick PCR purification kit (Qiagen). Purified His_6_-CpxR protein was phosphorylated with 50 mM acetyl phosphate (Sigma-Aldrich) in buffer containing 10 mM magnesium chloride for 1 h at 30°C. The PCR products were incubated with the CpxR-P protein (0 to 2 μM) in a binding buffer containing Tris-HCl pH 7.5 (20 mM), KCl (50 mM), dithiothreitol (1 mM), and 5% glycerol, for 20 min at room temperature, and then were electrophoretically separated in 6% nondenaturing polyacrylamide gels in 0.5% Tris-borate-EDTA buffer at 4°C. The DNA bands were stained with ethidium bromide and visualized under UV light.

### Intracellular replication assays.

RAW264.7 (ATCC TIB-71) mouse macrophages were seeded at a density of 10^6^ cells per well in 24-well tissue culture plates for 24 h. Bacteria were obtained from cultures in N-MM and were opsonized with normal mouse serum in RPMI containing 10% fetal bovine serum (FBS) for 30 min on ice. Bacteria were added to cells at a multiplicity of infection (MOI) of 100. Plates were centrifuged at 5,000 × *g* for 5 min at 4°C and incubated for 30 min at 37°C under a humidified 5% CO_2_ atmosphere. Cells were washed three times with 1× PBS and then were incubated in RPMI containing 100 μg/mL gentamicin and 10% FBS over 1 h to eliminate extracellular bacteria. After this time, the gentamicin concentration was decreased to 10 μg/mL, and cells were incubated for an additional 1 h. Infected macrophages were incubated at 37°C for 2 h and 18 h in a humidified atmosphere with 5% CO_2_. For enumeration of intracellular bacteria, macrophages were washed three times with 1× PBS and lysed with 0.1% Triton X-100 for 15 min. Then, 10-fold serial dilutions were plated onto LB agar plates, which were incubated overnight at 37°C. CFU were counted in the plates. The experiment was performed three times in triplicate. The replication index was obtained by dividing the number of CFU per milliliter at 18 h by the number at 2 h.

### Statistical analysis.

All data are means from three independent experiments. Statistical analysis was performed using Prism 8.0 software (GraphPad, Inc., San Diego, CA, USA). One-way analysis of variance (ANOVA) followed by Tukey’s multiple-comparison test and unpaired Student's *t* test was performed. *P* values of ≤0.05 were considered statistically significant.
